# Epidemiological features and sociodemographic factors associated with mumps in mainland China from 2004 to 2018

**DOI:** 10.1002/jmv.27955

**Published:** 2022-07-09

**Authors:** Xiaofang Fu, Minjie Ge, Wucheng Xu, Min Yu, Jiangang Ju, Yonghong Zhong, Huaqiong Huang

**Affiliations:** ^1^ Linping Campus The Second Affiliated Hospital of Zhejiang University School of Medicine Hangzhou China; ^2^ Key Laboratory of Respiratory Disease of Zhejiang Province, Department of Respiratory and Critical Care Medicine The Second Affiliated Hospital of Zhejiang University School of Medicine Hangzhou Zhejiang China

**Keywords:** epidemiology, mainland China, mumps, sociodemographic factors

## Abstract

Mumps is an acute infectious disease that spreads widely around the world. The aim of this study was to investigate the epidemiological features and sociodemographic factors associated with mumps in mainland China from 2004 to 2018. Incidence data for mumps during the period 2004–2018 were collected from the Public Health Sciences Data Center of China. Joinpoint regression analysis was performed to explore the trends of mumps. Space–time clustering analysis was conducted to spatial and temporal aggregation areas of mumps. A generalized linear model was used to explore sociodemographic factors associated with the incidence of mumps. The average annual incidence of mumps was 21.44/100 000 in mainland China. It was increased dramatically during 2004–2012 (annual percentage change​ [​​​​​​APC] = 7.51, 95% confidence interval [CI]: 2.28–13.00). After 2012, it remained stable, however, significantly increased in intermediately developed regions from 2015 to 2018 (APC = 25.84, 95% CI: 3.59–52.86). The first‐level spatial and temporal aggregation areas were distributed in Xinjiang, Gansu, Qinghai, Ningxia and Shaanxi, Tibet, Sichuan, Yunnan, Chongqing, Guizhou, and Guangxi, with gathering times from January 1, 2006 to December 31, 2012 (relative risk [RR] = 1.87, *p* < 0.001). The percentage of the population aged 0–14 years, number of health workers per capital, and number of passengers were found to be positively associated with the incidence of mumps. Overall, after 2012, the incidence of mumps in mainland China remained stable. High‐risk periods, clusters of regions, and sociodemographic factors for mumps were identified, which will help the government develop the disease‐ and location‐specific interventive measures.

**Abbreviations**
95% CI: 95% confidence interval
APC: annual percentage change
AAPC: average annual percentage change
COVID‐19: corona virus disease 2019
GLM: generalized linear model
LLR: log‐likelihood ratio
MMR: measles–mumps–rubella
MuV: mumps virus
RR: relative risk

## 1 BACKGROUND

Mumps is an acute respiratory infectious disease caused by the mumps virus, with a high incidence of prevaccine diseases.^1^ Mumps is characterized by mumps swelling and pain, sometimes straining other salivary glands, and common complications include viral encephalitis, pancreatitis, and ovaritis.^2^ In addition, some studies have found that mumps is a major cause of male infertility and acquired deafness in children.^3^, ^4^, ^5^ Because its clinical manifestation is mild, mumps has been somewhat neglected compared with other infectious diseases (e.g., measles and chickenpox).^6^ However, the incidence of mumps has been on the rise in recent years, especially in school‐age children and scattered children, which seriously affects children's health and quality of life. Large mumps outbreaks have occurred in the United States and several European countries in recent years.^3^, ^7^, ^8^, ^9^, ^10^ The number of mumps cases reported in the United States each year in 2016 and 2017 was nearly double the total number reported in the previous 5 years.^11^, ^12^ The number of mumps cases in China increased annually, ranking second only to influenza in the incidence of Class C infectious diseases from 2016 to 2019.^13^, ^14^ These data suggest that mumps remains a severe global public health problem.

In 2004, the National Notifiable Disease Reporting System of China CDC (NNDRS) was initiated at the China CDC and the number of reported mumps cases was collected for the entire country. Although previous studies on mumps in China described mumps‐associated epidemic estimates, these works were based on the region‐specific level or short‐term nature.^15^ Therefore, we used Joinpoint regression to describe the national trend and variation in mumps during 2004–2018. The surveillance data at the provincial level were analyzed by exploratory space–time clustering analysis. We performed a generalized linear model for the identification and estimation of the mumps risk. This study aimed to explore the trend variation, space–time clustering, and sociodemographic factors of mumps, which will provide data support to key groups and regions for prevention and control.

## 2 MATERIALS AND METHODS

### 2.1 Data sources

Incidence data of mumps from 2004 to 2018 were provided by the Public Health Sciences Data Center of China, which is a component of the national scientific data sharing platform of the national science and technology infrastructure in China and covers a population of approximately 1.3 billion people from 31 provinces and regions in mainland China.^16^ Mumps is classified as a Category C infectious disease in China. Health departments command that infected patients, suspected infection patients, or infectious pathogens must be reported online within 24 h once diagnosed or be reported by means of a network report card filled out by trained health personnel and sent within 24 h if the medical institutions do not have the conditions for direct reporting on the internet. All disease‐reporting data were reviewed by professionals to ensure their accuracy and reliability. The diagnosis of mumps cases was based on the “Diagnostic Criteria for Mumps” issued by the Ministry of Health.^17^ The diagnosis is not difficult according to the epidemic situation and exposure history, as well as the characteristics of parotid gland enlargement. In case of atypical suspicious cases, further diagnosis can be made by laboratory examination. A mumps case met at least one of the following criteria: (1) a history of close contact with mumps patients 1–4 weeks before the onset of the disease; (2) fever, chills, fatigue, loss of appetite, and after 1–2 days unilateral or bilateral non‐suppurative parotid swelling and pain or other salivary gland swelling and pain; the parotid duct orifice may appear redness; (3) laboratory examination: (I) white blood cell count normal or slightly low; (ii) serum amylase was slightly or moderately elevated in 90% of patients; (iii) serological and etiological tests can also be used as diagnostic evidence.

### 2.2 Statistical analysis

#### 2.2.1 Joinpoint regression

The Joinpoint regression model was used to examine the trends of incidence of AHC from 2004 to 2018 in mainland China. We further divided 31 provinces/municipalities/regions into three income groups (high‐, middle‐, low income) by its ranks of per capita gross domestic product in 2018. Joinpoint regression analysis fits a series of joined linear models of the natural logarithm of annual incidence using calendar year as an independent variable, which is often helpful in describing changes in trend data.^18^, ^19^ The joinpoint regression model for the observations ($x_{1}$, $y_{1}$),…, ($x_{n}$, $y_{n}$), where $x_{1}$ ≤…≤ $x_{n}$ without loss of generality, may be written as

$$Ε\left(y|x\right)=\beta_{0\;}\;\;+\beta_{1\;}x+{\delta_{1\;}\;\;(x-\tau_{1})}^{+}+\text{…}+{\delta_{\kappa }\left(x-\tau_{\kappa }\right)}^{+}$$

where y is the outcome of interest, x is the calendar year, $\beta_{1}$ is the regression coefficient, $\delta_{\kappa }$ represents the regression coefficient of the piecewise function in section *k*, $\tau_{\kappa }$ is the unknown joinpoints; $\alpha =\left(x-\tau_{\kappa }\right),\;\alpha$ ^+^= α for α > 0 and 0 otherwise.

We allowed a maximum of five joinpoints for estimation, as recommended by the program developer, and used Bayesian information criterion (BIC) to select the best‐fitted model. The annual percentage changes (APCs) with their 95% confidence interval (CI) were expressed for each trend segment and average annual percentage change (AAPC) with their 95% CI were expressed for annual mean change. Once the unknown joinpoints $\varepsilon_{n}\;$was determined, we estimate the APCs of each period segment; the APC is calculated as ${\mathrm{APC}}_{i}=[(\mathrm{Εxp}(\beta_{i})-1)]\times 100$, where $\beta_{i}$ represents the slope of the period segment. The *Z* test was used to assess whether APCs were significant (*p* < 0.05), and the trends were further described as increased or decreased when the APCs were positive or negative, respectively, while the trends were considered as stable when the APCs values were not significant (*p* < 0.05). Joinpoint regression analysis was conducted with Joinpoint regression software (version 4.8.0.1, National Cancer Institute).

#### 2.2.2 Space–time cluster analysis

Space–time cluster analysis was performed based on the discrete Poisson model at the provincial level using SaTScan software (version 9.7).^20^ The software is used to detect spatiotemporal transmission aggregation patterns of specific diseases, find abnormal increased or decreased incidence between research units (usually the regional distribution and incidence of study subjects), and through hypothesis test to determine whether abnormal phenomena result from random. The basic principle is to set the scanning window to traverse scanning in space and time. Generally, the mobile window method is used to establish a three‐dimensional dynamic cylindrical window in the research area. The bottom area represents the area and the height represents the duration. When the incidence within the cylinder is found to be higher than outside the cylinder, the cylinder is considered a spatio‐temporal aggregation event, a potential outbreak. The statistical value was the log‐likelihood ratio (LLR), with a larger LLR value indicating a more likely gathering area. The relative risk (RR) was also calculated. Finally, the result was visualized through ArcMap software (version 10.2).

#### 2.2.3 Generalized linear model

We used the log‐linear generalized linear model to fit the incidence of mumps and sociodemographic factors covariates during 2004–2018. The details are as follows:

$$\ln(\text{incidence of mumps})=\beta_{0}+\beta_{1}*\;\text{Year}\;+\;\beta_{2}*\;\text{percentage of population aged}\;0-14+\beta_{3}*\;p\text{ercentage of urban population}+\beta_{4}*\;\text{unemployment rate}+\beta_{5}\;*\;\text{proportion of population at college and above}+\beta_{6}*\;\ln(\text{gross domestic product}(\text{GDP}))+\beta_{7}*\ln(\text{number of passengers})+\beta_{8}*\ln(\text{healthcare workers per capital})+\beta_{9}*\;\ln(\text{turnover of passenger traffic}(100\;\text{million passenger}\;-\;\text{kilometer}))+\beta_{10}*\;\ln(\text{number of medical institutions})+\beta_{11}*\;\ln(\text{population density})+\beta_{12}*\;\ln(\text{lengths of highways}(10\;000\;\text{km}))+\beta_{13}*\;\ln(\text{lengths of railways}(10\;000\;\text{km}))$$

We determined the best‐fitting model according to the Akaike information criterion.

## 3 RESULTS

### 3.1 The incidence of mumps in mainland China from 2004 to 2018

**Figure 1:**
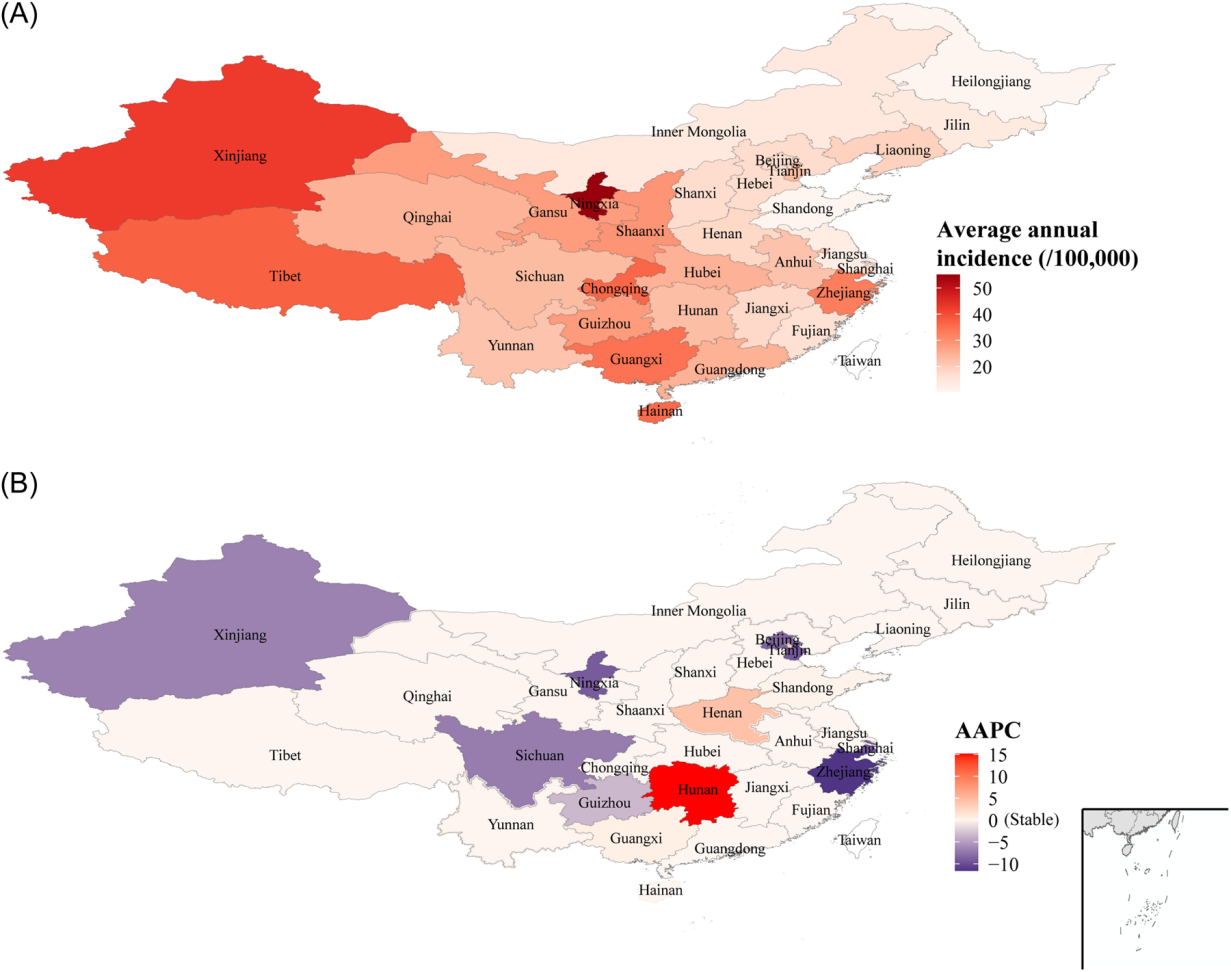
The incidence of mumps in mainland China from 2004 to 2018. (A) The average annual incidence of mumps from 2004 to 2018. (B) The AAPC of mumps from 2004 to 2018.

A total of 4 269 946 mumps cases were reported in mainland China from January 1, 2004 to December 31, 2018. The average annual incidence was 21.26/100 000, ranging from 10.33/100 000 to 55.12/100 000 (Table 1). The highest mumps incidences were mainly distributed in northwestern and central China, such as Ningxia, Xinjiang, Tibet, Chongqing, Hainan, Guangxi, and Zhejiang, with the incidences exceeding 30/100 000. Among them, Ningxia and Xinjiang possessed considerably higher incidences, with values of 55.12/100 0000 and 42.45/100 0000, respectively (Figure 1A and Table 1). Two provinces showed significant increasing trends during our study period, including Hunan (AAPC = 15.15, 95% CI: 0.99–31.28, *p* < 0.05) and Henan (AAPC = 4.25, 95% CI: 1.03–7.57, *p* > 0.05). In other provinces, the incidence of mumps showed a stable or decreased state during our study period (Figure 1B and Table 1).

**Table 1:** The cases of mumps in mainland China from 2004 to 2018

Region	Number of cases	Average annual incidence (/100 000)			AAPC (95% CI)
		2004–2018	2004–2012	2013–2018	
Ningxia	51 527	55.12	69.99	32.81	−9.11 (−17.17 to −0.26)*
Xinjiang	136 093	42.45	52.8	26.93	−6.68 (−11.16 to −1.98)*
Tibet	16 315	37.33	53.07	13.7	−9.48 (−19.92 to 2.31)
Chongqing	159 181	36.34	39.87	31.05	−4.46 (−14.38 to 6.60)
Hainan	47 659	35.75	35.43	36.22	15.10 (−7.8 to 43.69)
Guangxi	246 319	34.71	41.95	23.86	0.39 (−12.86 to 15.66)
Zhejiang	252 860	32.8	44.71	14.92	−11.60 (−15.05 to −8.01)*
Shaanxi	163 992	29.17	33.48	22.69	−1.33 (−6.86 to 4.53)
Guizhou	153 927	28.19	32.62	21.56	−3.45 (−6.7 to −0.08)*
Gansu	108 106	27.81	33.26	19.63	1.32 (−22.02 to 31.63)
Guangdong	371 464	24.7	28.34	19.23	−1.11 (−6.12 to 4.16)
Hubei	213 306	24.66	25.97	22.69	9.81 (−2.8 to 24.05)
Qinghai	20 639	24.42	25.67	22.55	−2.02 (−7.12 to 3.36)
Tianjin	40 748	23.11	31.53	10.47	−9.28 (−16.92 to −0.94)*
Sichuan	282 666	22.96	28.7	14.33	−6.96 (−10.41 to −3.38)*
Hunan	223 861	22.54	18.05	29.28	15.15 (0.99–31.28)*
Anhui	199 470	21.85	22.15	21.39	5.17 (−8.42 to 20.77)
Yunnan	149 182	21.68	26.15	14.98	13.97 (−3.43 to 34.49)
Liaoning	124 418	19.23	26.77	7.92	−7.00 (−16.59 to 3.70)
Shanghai	53 318	18.2	23.2	10.69	−9.34 (−10.92 to −7.73)*
Jiangxi	120 400	18.03	19.61	15.66	−2.81 (−14.9 to 10.99)
Henan	254 780	17.97	16.54	20.12	4.25 (1.03 to 7.57)*
Beijing	47 102	17.68	22.18	10.93	−8.53 (−9.67 to −7.37)*
Hebei	185 237	17.29	19.76	13.59	3.04 (−12.9 to 21.90)
Shanxi	90 860	17.17	18.9	14.56	1.02 (−4.13 to 6.44)
Fujian	91 047	16.54	21.56	9.01	−5.27 (−11.64 to 1.56)
Inner Mongolia	51 786	14.04	15.44	11.94	1.09 (−11.48 to 15.45)
Jilin	55 973	13.64	16.86	8.8	0.92 (−8.36 to 11.13)
Jiangsu	149 302	12.82	14.06	10.95	−3.63 (−8.42 to 1.41)
Shandong	149 183	10.39	10.69	9.94	−0.10 (−6.05 to 6.22)
Heilongjiang	59 225	10.33	13.2	6.03	−4.41 (−15.22 to 7.77)
Mainland China	4 269 946	21.26	24.2	16.87	0.70 (−8.31 to 10.58)

* Values are significance *p* < 0.05.

### 3.2 The seasonal and age group distribution of mumps from 2004 to 2018

**Figure 2:**
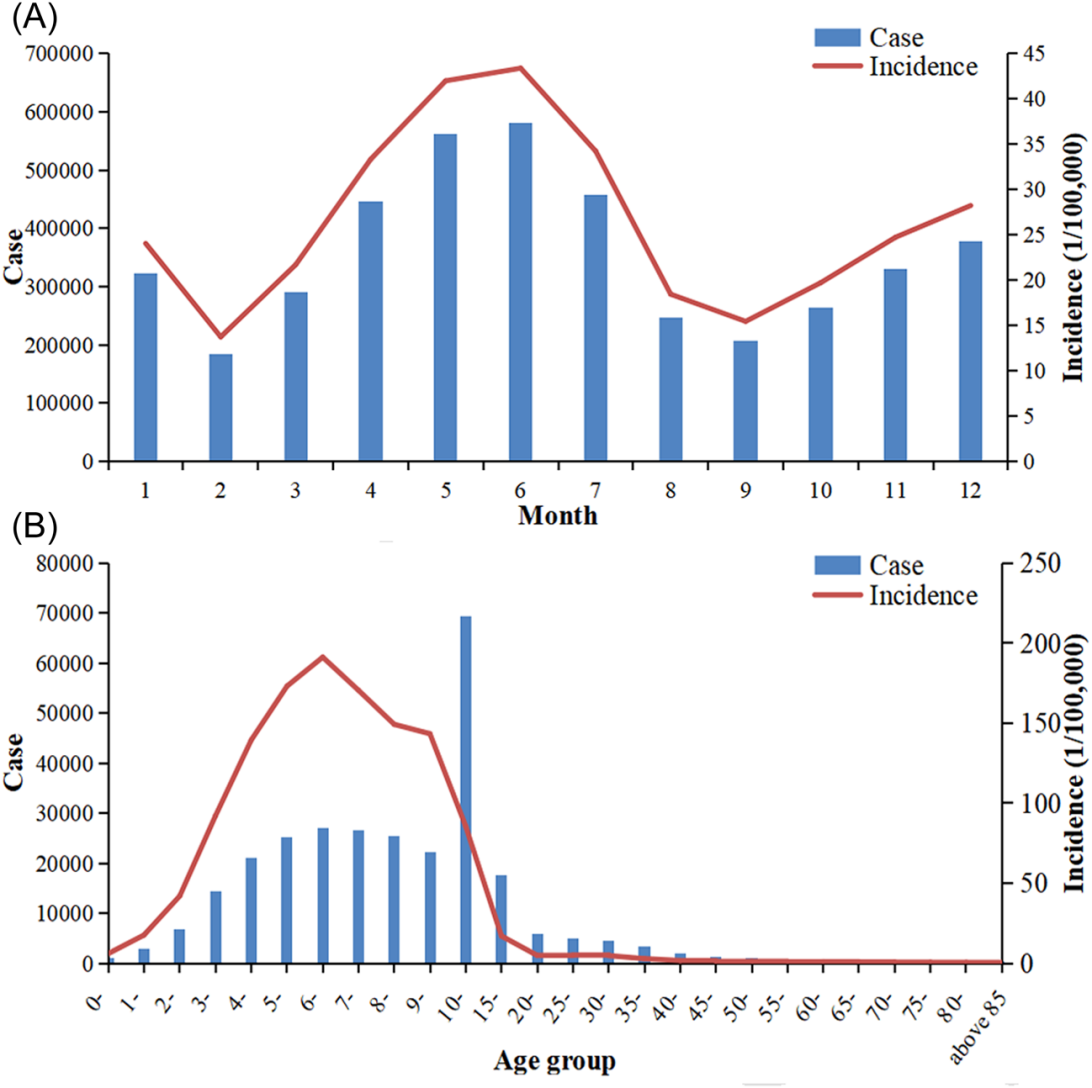
The seasonal and age group distribution of mumps from 2004 to 2018. (A) The seasonal distribution of mumps from 2004 to 2018. (B) The seasonal and age group distribution of mumps from 2004 to 2018.

Mumps cases were highly influenced by season during 2004–2018. The two peak periods of mumps are April to July and December to January of the next year, with a fluctuation range of 24.05/100 000 and 43.38/100 000, respectively (Figure 2A). According to age group, the age group of 6‐year age showed the highest average annual incidence of 191.27/100 000. Children in the 1–15‐year group were the most at‐risk group infected with mumps, accounting for 83.62% of all cases. With increasing of age, the incidences showed a downward trend, and after >15 years, the age group showed lower mumps case numbers (Figure 2B).

### 3.3 The trends of incidence of mumps at different economic levels from 2004 to 2018

**Figure 3:**
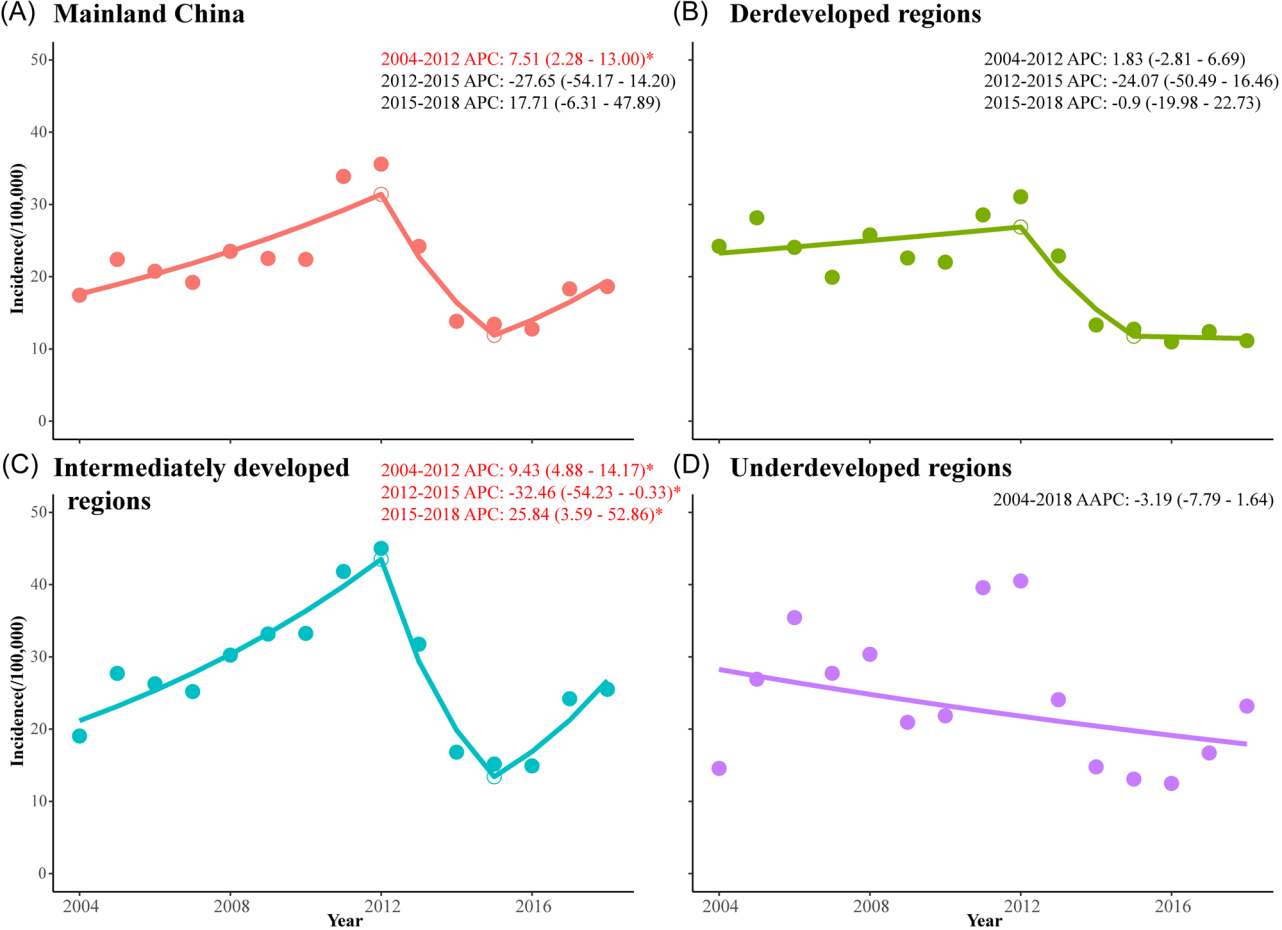
The trends of incidence of mumps at different economic levels from 2004 to 2018. (A) The trends of incidence of mumps in mainland China from 2004 to 2018. (B) The trends of incidence of mumps in developed regions from 2004 to 2018. (C) The trends of incidence of mumps in intermediately developed regions from 2004 to 2018. (D) The trends of incidence of mumps in undeveloped regions from 2004 to 2018.

The results of Joinpoint analysis showed that overall, the incidence of mumps was stable in mainland China from 2004 to 2018 (AAPC = 0.70, 95% CI: −8.31 to 10.58, *p* > 0.05). The incidence of mumps showed a significantly increasing trend from 2004 to 2012, with an APC of 7.51 (95% CI: 2.28–13.00, *p* < 0.05) and an incidence of 69.99/100 000 peaked in 2012. Then the incidence of mumps remained stable during 2013–2018 (Figure 3A).

The intermediately developed regions in particular showed a rapid rise from 2004 and reached a peak in 2012, with the most cases reported (APC = 9.43, 95% CI: 4.88–14.17, *p* < 0.05), followed by a significantly decreasing trend with the lowest level in 2015 (APC = −32.46, 95% CI: −54.23 to −0.33, *p* < 0.05) and a slight increase afterward (APC = 25.84, 95% CI: 3.59–52.86, *p* < 0.05) (Figure 3C). Overall, the mumps incidence remained steady in the developed and underdeveloped regions (Figure 3B,D).

### 3.4 Space–time clustering analysis of mumps in mainland China from 2004 to 2018

**Figure 4:**
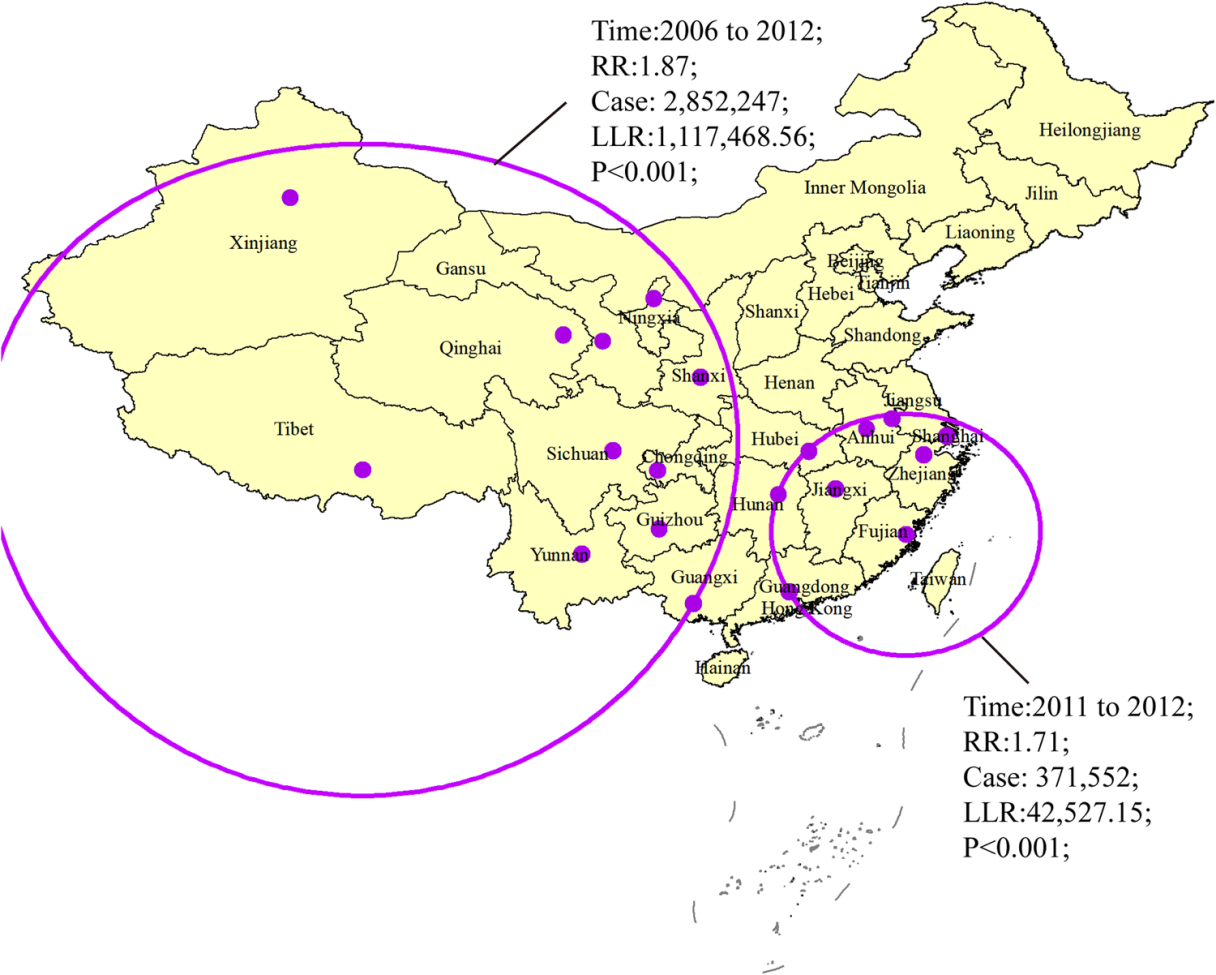
Space–time cluster of mumps in mainland China from 2004 to 2018

Two spatial and temporal aggregation areas of mumps incidence were revealed in mainland China from 2004 to 2018. The first‐level spatial and temporal aggregation areas were distributed in northwest China (Xinjiang, Gansu, Qinghai, Ningxia, and Shaanxi), southwest China (Tibet, Sichuan, Yunnan, Chongqing, and Guizhou), and south China (Guangxi), with the gathering times from January 1, 2006 to December 31, 2012. The actual number of cases reported in the regions was 852 247, which higher than expected, that was 502 210 (RR = 1.87, LLR = 1 117 486.56, *p* < 0.001). Furthermore, the secondary spatial and temporal aggregation areas covered eight provinces in southeast China (including Guangdong, Hunan, Jiangxi, Anhui, Jiangsu, Shanghai, Zhejiang, and Fujian) from January 1, 2011 to December 31, 2012. The actual number of cases reported in the regions was 371 552, which higher than expected, that was 224 963 (RR = 1.71, LLR = 42 527.15, *p* < 0.001; Figure 4).

### 3.5 Sociodemographic factors associated with the incidence of mumps

In the generalized linear model, the percentage of the population aged 0–14 years (*β* = 4.82, *p* < 0.001), number of health workers per capital (*β* = 0.73, *p* < 0.001), and number of passengers (*β* = 0.24, *p* < 0.001) were found to be positively associated with the incidence of mumps, while the proportion of the population at college and above (*β* = −1.79, *p* = 0.039), gross domestic product (*β* = −0.12, *p* < 0.001), population density (*β* = −0.15, *p* < 0.001), length of highways (*β*= −0.19, *p* =  0.043) and railways (*β* = −0.40, *p* < 0.001) presented a negative correlation (Table 2).

**Table 2:** Sociodemographic factors associated with incidence of mumps

Variables	*β*	SE	*t* value	*p*
Intercept	29.51	23.94	1.23	0.218
Year	−0.01	0.01	−1.07	0.287
Percentage of population aged 0–14	**4.82**	**1.25**	**3.85**	**<0.001**
Percentage of urban population	0.16	0.62	0.25	0.803
Unemployment rate	0.07	0.05	1.41	0.158
Proportion of population at college and above	**−1.79**	**0.86**	**−2.07**	**0.039**
Log gross domestic product	**−0.12**	**0.03**	**−4.09**	**<0.001**
Log number of medical institutions	0.11	0.07	1.50	0.136
Log number of Health worker (/10 000)	**0.73**	**0.20**	**3.65**	**<0.001**
Log number of passengers	**0.24**	**0.06**	**4.16**	**<0.001**
Log population density	**−0.15**	**0.04**	**−3.63**	**<0.001**
Log turnover of passenger traffic (100 million passenger‐kilometer)	0.14	0.08	1.76	0.079
Log lengths of highways (10 000 km)	**−0.19**	**0.09**	**−2.02**	**0.043**
Log lengths of railways (10 000 km)	**−0.40**	**0.07**	**−5.59**	**<0.001**

*Note*: Bold values are significant *p* < 0.05.

## 4 DISCUSSION

Over the past decades, infectious disease outbreaks have been on the rise every year,^21^ and we have to be on guard against outbreaks of other viruses in the global Corona Virus Disease 2019 (COVID‐19) pandemic. Mumps virus is mainly transmitted from person to person through air droplets, regardless of climate restrictions, so the region is very widely distributed and has periodic outbreaks. Our analysis found that the average annual incidence of mumps varies greatly among different provinces, and the top three regions with the highest incidence rates were Ningxia, Xinjiang, and Tibet. Significant spatial–temporal aggregation areas of mumps were detected during our study period. The clustering regions were mainly distributed in Southwest China, Northwest China, and Southeast China. We also found that the epidemic pattern of mumps varies with different economic levels. The incidence of mumps in the intermediately developed regions fluctuated greatly, while the incidence of the disease at the low economic level showed a steady pattern. The findings of our study can provide a basis for targeted and effective measures for controlling mumps in China.

Mumps have strong infectivity, a recessive infection rate is high, and the infection period is long, leading to early patients and recessive infection patients who are not easy to be find, easily leading to the outbreak of the disease. In 2008, the measles–mumps–rubella (MMR) combination vaccine was included in the national immunization program,^22^ and the mumps vaccination rate of school‐age children was required to reach over 90% before 2010 in China. The morbidity of mumps in children aged 0–14 years showed a significant downward trend in Zhejiang Province, Beijing, Tianjin, and Shanghai after 2008.^23^, ^24^ However, compared with before vaccines were included in the national immunization program, the incidence of mumps in northwest China did not change significantly from 2008 and even peaked in 2011–2012. The potential reasons for this may be related to the following factors. Firstly, the level of immunization coverage is an important indicator to determine the number of susceptible people. Mumps vaccines are routinely performed in more than 120 countries and have resulted in a distinct decrease in mumps incidence. Due to the difference in vaccine supply and economic level, the vaccination rates of MuV in China vary greatly. In Gansu Province, routine immunization with mumps vaccine from 2010 to 2012 was insufficient, and children of appropriate age were not vaccinated in time, with a vaccination rate of less than 30%.^25^ However, the first dose MMR coverage had remained above 95% since the 1999 birth cohort and the second dose MMR coverage reached above 90% since the 2006 birth cohort in Shanghai.^26^ Global experience shows that the prevention and control of mumps requires a sustained high level of immunization coverage and >1 dose immunization program.^27^ Secondly, the 2011–2012 mumps epidemic may also be due to the accumulation of many mumps‐susceptible individuals, as mumps vaccine was introduced into national immunization programs for only 3–4 years and they were not vaccinated against mumps during 2011–2012. Therefore, the mumps vaccine had no effect on the 2008–2012 Chinese mainland mumps outbreak. Finally, the level of medical care is relatively inadequate and primary medical institutions are not strongly aware of epidemic reporting, which are nonnegligible reasons for the spread of mumps epidemics in economically underdeveloped areas.^28^ These reasons suggested that the first and most basic action is to improve vaccination coverage and enhance disease surveillance, especially in northwest China.

The incidence of mumps in China showed a relatively stable trend after 2012, but mumps incidence in intermediately developed areas exhibited a significant increasing trend from 2015 to 2018. The reasons for the rising incidence of mumps in intermediately developed regions from 2015 to 2018 need to be further explored. On the one hand, with the implementation of the reform and opening up policy and the rapid development of the southeast coastal areas, a large number of people from underdeveloped areas have flocked to intermediately developed regions or developed cities to seek job opportunities, and many children have migrated irregularly with their parents, making it difficult to fully implement the national immunization program in this group. The accumulation of susceptible children who are brought together in high‐density settings leads to a high force of infection and increased risk of exposure. On the other hand, although in previous studies it was considered that the estimated effectiveness of the two doses of the MMR vaccine was between 66% and 95%,^29^, ^30^, ^31^, ^32^, ^33^ the recent mumps outbreak in individuals who received two doses of MMR vaccine has raised challenges to the effectiveness.^34^, ^35^ Mumps epidemics have re‐emerged in many countries in recent years among highly immunized adolescents and young adults due to inadequate vaccine effectiveness, waning immunity, periodicity of the disease or genetic divergences of vaccine and wild‐type strains.^29^, ^36^ Previous results showed that three doses of MMR vaccine can effectively improve the immune effect of mumps antibody.^37^, ^38^, ^39^ Currently, the immunization programs of two doses of MMR are given at the age of 8 and 18 months in China.^40^ To further reduce the incidence of mumps and prevent the resurgence of the epidemic situation, immunization with three doses of the MMR vaccine has been carried out in many developed areas in China. However, the effect of a third dose of the MMR vaccine in stemming a mumps outbreak is unknown.

The significant space–time clustering analysis of mumps in our study was detected during our study period. The clustering regions were distributed in northwestern China, southwestern China, and southeastern China, and the clustering period was 2006–2012 and 2011–2012, respectively, which is in accord with that of joinpoint regression. Ningxia, Xinjiang, and especially Tibet were previously the most seriously affected epidemic provinces. The epidemic in the northwest and southwest may have a lot to do with the low coverage of mumps vaccine in 2004–2012. Moreover, the coastal high‐risk areas in different studies generally exhibited the same problems, such as large migrant floating populations, relatively high population densities, and poor‐quality dwellings. It may be the cause of clusters. The government should pay more attention to high‐risk areas in China.

Our study showed that sociodemographic variables such as the number of people aged 0–14, proportion of the population at college and above, gross domestic product (GDP) level, number of health workers per 10 000, number of passengers, length of highways, and length of railways were all significantly associated with the incidence of mumps. It is interesting to note that the incidence of mumps was negatively correlated with the lengths of highways and the lengths of railways. Theoretically, the more developed highways and railways are, the greater the risk of infectious diseases as a result of the higher mobility of people. Nevertheless, the economic level of areas with developed railways and highways is generally above the medium level, and the GDP level, medical level, number of health workers per capita, and coverage rate of mumps vaccination are higher. Previous studies have shown that the level of GDP and the number of health‐care facilities can help control infectious diseases.^41^, ^42^ The allocation of adequate health resources and increased coverage of immunization can remarkably reduce the population's susceptibility to mumps and strengthen the ability to control the epidemic.^43^ In addition, a lower educated population may increase the risk of infections in cold environments, which may be related to the poor living conditions and the weak health awareness of mumps incidence.^43^ Regrettably, our study did not assess the impact of climate on the incidence of mumps. Unlike previous studies,^43^ our study showed a negative correlation between population density and mumps incidence. These findings may need further extensive studies for confirmation.

## 5 CONCLUSIONS

In summary, during the past decade, the incidence of mumps was stable in mainland China. However, it was heavy in western China and significantly increased in the intermediately developed regions from 2015 to 2018. Regions with a high percentage of the population aged 0–14 years and a high number of passengers are at high risk of mumps incidence. In the future, improvement of vaccine coverage and public health policy priorities for mumps in the regions with these characteristics are necessary.

## AUTHOR CONTRIBUTIONS

*Data curation*: Xiaofang Fu, Minjie Ge, Wucheng Xu, Min Yu, Jiangang Ju, and Yonghong Zhong. *Data analysis*: Xiaofang Fu and Minjie Ge. *Writing—original draft*: Xiaofang Fu. *Supervision, writing—review & editing*: Huaqiong Huang.

## CONFLICT OF INTEREST

The authors declare no conflict of interest.

## Data Availability

The data that support the findings of this study are openly available in Public Health Sciences Data Center of China at https://www.phsciencedata.cn/Share/.
